# Low serum gastrin associated with ER^+^ breast cancer development via inactivation of CCKBR/ERK/P65 signaling

**DOI:** 10.1186/s12885-018-4717-7

**Published:** 2018-08-16

**Authors:** Li-Li Meng, Jing-Long Wang, Shu-Ping Xu, Li-Dong Zu, Zhao-Wen Yan, Jian-Bing Zhang, Ya-Qin Han, Guo-Hui Fu

**Affiliations:** 1 Pathology Center, Shanghai General Hospital/Faculty of Basic Medicine, Shanghai Jiao Tong University School of Medicine, No. 280, South Chong-Qing Road, Shanghai, 200025 People’s Republic of China; 2 Key Laboratory of Cell Differentiation and Apoptosis of Chinese Ministry of Education, Institutes of Medical Sciences, Shanghai Jiao Tong University School of Medicine, Shanghai, China; 3 Shanghai Key Laboratory of Gastric Neoplasms, Shanghai Institute of Digestive Surgery, Ruijin Hospital, Shanghai Jiao Tong University School of Medicine, Shanghai, China; 4 Breast Surgery Division, Zhuhai Hospital of Integrated Traditional Chinese and Western Medicine, Zhuhai, China

**Keywords:** Gastrin, Breast cancer, ER, ERK, P65

## Abstract

**Background:**

Gastrin is an important gastrointestinal hormone produced primarily by G-cells in the antrum of the stomach. It normally regulates gastric acid secretion and is implicated in a number of human disease states, but how its function affects breast cancer (BC) development is not documented. The current study investigated the suppressive effects of gastrin on BC and its underlying mechanisms.

**Methods:**

Serum levels of gastrin were measured by enzyme-linked immunosorbent assay (ELISA) and correlation between gastrin level and development of BC was analyzed by chi-square test. Inhibitory effects of gastrin on BC were investigated by CCK-8 assay and nude mice models. Expressions of CCKBR/ERK/P65 in BC patients were determined through immunohistochemistry (IHC) and Western blot. Survival analysis was performed using the log-rank test.

**Results:**

The results indicated that the serum level of gastrin in BC patients was lower compared with normal control. Cellular and molecular experiments indicated that reduction of gastrin is associated with inactivation of cholecystokinin B receptor (CCKBR)/ERK/P65 signaling in BC cells which is corresponding to molecular type of estrogen receptor (ER) positive BC. Furthermore, we found that low expression of gastrin/CCKBR/ERK /P65 was correlated to worse prognosis in BC patients. Gastrin or ERK/P65 activators inhibited ER^+^ BC through CCKBR-mediated activation of ERK/P65. Moreover, combination treatment with gastrin and tamoxifen more efficiently inhibited ER^+^ BC than tamoxifen alone.

**Conclusions:**

We concluded that low serum gastrin is related to increased risk of ER^+^ BC development. The results also established that CCKBR/ERK/P65 signaling function is generally tumor suppressive in ER^+^ BC, indicating therapies should focus on restoring, not inhibiting, CCKBR/ERK/P65 pathway activity.

**Electronic supplementary material:**

The online version of this article (10.1186/s12885-018-4717-7) contains supplementary material, which is available to authorized users.

## Background

Breast cancer (BC) is the most common form of female cancer worldwide. Gene expression profiling has a considerable impact on the current understanding of BC biology and has led to improved treatment outcomes [1–3]. Studies have extensively characterized molecular subtypes of BC, which are clinically subdivided as hormone receptor-positive, human epidermal growth factor receptor 2 (HER2)-positive, and triple-negative BC (TNBC) [4–6]. These subtypes have significantly different incidence, risk factors, prognoses, and treatment sensitivities [7, 8]. During the past few decade years, most great advances had been made in molecular biology and clinical treatments by utilizing a combination of surgical resection with radiotherapy, chemotherapy, and targeted therapy to cure breast cancer. Although the comprehensive treatment involving the mammography [9] and HER2 status [10] has a certain effect on screening and diagnosis, the recurrence and metastasis rates for breast cancer patients remain high. The poor clinical outcome of breast cancer was attributed to the metastasis and invasion. Among the matrix metalloproteinases (MMPs), a family of zinc endopeptidases with the role on extracellular matrix protein degradation leading to the metastasis of cancer cells, specifically, the serum activities of MMP-2 and MMP-9 correlate with the invasive potential of cancer [11].

Approximately 70% of BCs shows the ER-α expression and endocrine responses, and hormonal therapy has produced a considerable amount of positive outcomes in ER^+^ BC [8, 12–14]. Among the agents applied in clinical cancer therapeutics, tamoxifen is one of the most successful agents that target ER [15–17]. Nonetheless, a large proportion of patients developed de novo or acquired resistance over time, and annually estimated deaths caused by BC are more than 450,000 worldwide [18, 19]. The most plausible explanation for this statistic shows the lack of a complete profile of BC heterogeneity. Even though main clinical parameters and pathological markers, such as ER, progesterone receptor (PR), and HER2, are not able to fully reflect the complexity of BC, they are routinely used in the clinic to stratify patients for prognostic predictions, treatment selection, and clinical trials [15, 20, 21]. Thus, more precise predictive biomarkers and optimal treatment strategies require the further investigation.

Recently, a study demonstrated the alteration of molecular markers in BC after clinical treatment [22], which suggested that molecular subtypes could be affected by the factors that penetrated the tumor microenvironment via blood circulation. Thus, genetic variations and the factors within the tumor microenvironment might cooperatively determine the molecular subtype.

Gastrin was initially characterized as the major hormonal regulator of gastric acid secretion [23–26]. Another physiologic role of gastrin involves regulating proliferation of gastric mucosal cells, which leads to investigations into its effects on stimulation of tumor cell growth [27–29]. However, its inhibitory effects have also been observed on several types of cancers derived from the colon, stomach, and pancreas [30–32]. These controversial reports have suggested that gastrin might play a role in organ- and/or molecular subtype-dependent manner.

CCKBR, a seven-transmembrane, G protein-coupled receptor is highly expressed in the proximal stomach, where the role on acid secretion is well documented. Previous report had shown that CCKBR knockout (CCK2R−/−) resulted in inactivation of MAPK pathway, especially the ERK1/2 [33]. In health cell lines, CCKBR and MOR heteromerization could also regulate the activity of ERK pathway [34]. Once the level of p-ERK was elevated, p-ERK phosphorylated P65 which resulted in P65 protein stability and the activation of downstream genes and factors involved in different cellular process. It is highly reasonable to speculate CCKBR/ERK/P65 cascade may play a role in breast cancer development. In this paper, we analyzed the expression level of CCKBR/ERK/P65 cascade and determined its effect on ER positive BC.

## Methods

### Clinical cases

All 93 BC cases, female and age ranging from 20 to 70 years (mean = 46.2 years), were collected from Shanghai General Hospital (Shanghai, China) and Zhuhai Hospital of Integrated Traditional Chinese and Western Medicine (Zhuhai, China) in 2016 (detail in Additional file 1: Table S2). All necessary BC patient information was available, including tumor characteristics (grade, lymph node stage, and size). Not all BC patients received chemo-radiotherapy before enrollment. Normal, healthy female volunteers who received a physical examination at Shanghai General Hospital were used as controls for gastrin measurement (*N* = 20). All patients gave verbal informed consent before inclusion. Approval from Ethics Committee of the Shanghai Jiao Tong University School of Medicine was obtained after they reviewed the study protocol and purpose.

### DFS and OS

Patients were given a physical examination every 3 months for the first 2 years after surgery and were subsequently examined every 6 months. Disease-free survival (DFS) was calculated as the duration between the date of surgery and the date of first evidence of local recurrence, distant metastasis, or diagnosis of a second primary tumor or cancer-associated mortality. Overall survival (OS) was calculated as the time between the date of surgery and the date of mortality from any cause.

### Blood collection and measurement of serum gastrin.

A 5-ml fasting venous blood sample was collected from 20 control and 93 BC subjects in the morning. The blood samples were immediately collected into endotoxin- and pyrogen-free test tubes, shaken thrice, and left to coagulate for 30 min at room temperature. Blood samples were centrifuged at 1000 x *g* for 10 min at 4 °C, then the serum was transferred to Eppendorf tubes and stored at − 80 °C until analysis. Gastrin levels in the serum were measured with a gastrin-17 ELISA kit (Sino Biological Inc., Beijing, China) in the same aliquot according to the manufacturer’s instructions while blinded to the histopathological diagnosis.

### Mouse models

Female athymic BALB/C nude mice (4–6 weeks-old) were purchased from the Shanghai Experimental Animal Center at the Chinese Academy of Science (Shanghai, China). BC-bearing mice were given estrogen subcutaneously 3 d before injecting MCF-7 cells (1 × 10^7^) into the bilateral mammary fat pad. These mice were then randomly divided into control (*N* = 6) and experimental (*N* = 6) groups when tumors became outwardly visible (approximately ≥10 mm^3^). Control and experimental groups received PBS or gastrin treatment (2 mg/kg, twice/d), respectively, by caudal vein injection for 12 d; tumor volume was measured every other day. At the end of the study period, mice were sacrificed by carbon dioxide euthanasia and tumors were removed, measured, weighed, and prepared for Western blot analysis. Tumor volume was calculated according to the formula: V (mm^3^) = (π x length x width^2^) / 6. All animal studies were conducted with the approval and guidance of Shanghai Jiao Tong University Medical Animal Ethics Committees.

### Cell culture and reagents

All human BC cell lines (MCF-7, T-47D, and MDA-MB-231) used in the study were purchased from the Cell Bank of Shanghai Institute of Cell Biology at the Chinese Academy of Science in 2016. The three cell lines were authenticated in Ministry of public security Evidence Identification Center by testing the DNA profiling (STR) entrusted by Shanghai Institute of Cell Biology in 2012 and 2013 respectively. The testing results were consistent with the profiles reported in ATCC. Mycoplasma detection was performed using MycAwayTM–color one step Mycoplasma detection Kit (Shanghai YEASEN Biotechnology Co., Ltd.) according to the manufacturer’s instructions. Cells were routinely cultured in Dulbecco’s modified Eagle’s medium (DMEM; Hyclone, USA) supplemented with 10% fetal bovine serum (Hyclone, USA) and incubated in air with 5% CO_2_ at 37 °C. In addition, the culture media for MCF-7 and T-47D were not supplemented with estradiol. Antibodies against ERK1/2 (monoclonal, 4695S, 137F5, CST, USA), phoshorylated ERK1/2 (p-ERK1/2) (monoclonal, 4370S, D13.14.4E, CST, USA), P65 (monoclonal, 8242S, D14E12, CST, USA), phoshorylated P65 (p-P65) (monoclonal, 3033S, 93H1, CST, USA), CCKBR (polyclonal, SC33221, Santa Cruz, USA), HER2 (monoclonal, GT210029, sp3, Gene Tech, Shanghai, China), ER (monoclonal, GT210029, sp1, Gene Tech, Shanghai, China), and PR (monoclonal, GT210029, sp2, Gene Tech, Shanghai, China) were used as primary antibodies for Western blot or/or immunohistochemistry (IHC). Gastrin was purchased from China Peptides (Shanghai, China). Lipopolysaccharides (LPS), PD98059, betulinic acid (BA), and parthenolide (PN) were purchased from Sigma Chemical Co. (St. Louis, Mo, USA). Cell proliferation assay was performed using Cell Counting Kit-8 (CCK-8; Dojindo, Japan).

### Protein extraction

Protein was extracted from cells and tissues with RIPA lysis buffer (50 mmol/l Tris-HCl [pH 7.5], 150 mmol/l NaCl, 0.5% DOC, 1% NP-40, 0.1% sodium dodecyl sulfate, 1 mmol/l NaF, 1 mmol/l Na_3_VO_4_, and 1 mM PMSF [Cell Signaling Technologies, Danvers, MA, USA]) purchased from Yeasen Biotech before being centrifuged at 12,000 x *g* for 20 min at 4 °C. The protein concentrations were determined by a BCA kit (Thermo Fisher Scientific, USA). Protein lysates were applied to Western blot.

### Western blot

Equal amount of proteins were separated on 10% sodium dodecyl sulfate –polyacrylamide gel electrophoresis and then transferred onto polyvinylidene fluoride membranes (Millipore, Billerica, MA, USA). Membranes were blocked in 5% bovine serum albumin (Amresco, USA) with shaking for 1 h at room temperature, and then washed in 1X Tris-buffered saline-Tween-20 (TBST) buffer thrice for 5 min each. Membranes were incubated overnight at 4 °C with anti-CCKBR (1:200), anti-ERK (1:1000), anti-p-ERK (1:1000), anti-P65 (1:1000), and anti-p-P65 (1:1000) primary antibodies. Then, membranes were washed thrice for 5 min each with 1X TBST and incubated with horseradish peroxidase-conjugated goat anti-rabbit or goat anti-mouse IgG secondary antibodies (Cell Signaling Technologies, Danvers, MA, USA) for 1 h at room temperature. Membranes were washed thrice for 5 min each with 1X TBST, and antigen-antibody complexes were visualized with a chemiluminescent ECL detection system (Pierce, Rockford, IL, USA). Blots were scanned and analyzed via Image J software.

### Cell proliferation assay

CCK-8 assay was performed to evaluate cell proliferation. BC cell lines (MCF-7, T-47D, and MDA-MB-231) were seeded onto 96-well plates at a density of 2 × 10^3^ cells/well, cultured, and treated with gastrin (10^− 7^ M), lipopolysaccharides (1 μg/ml), PD98059 (10 μM), betulinic acid (10 μg/ml), or parthenolide (10 μg/ml) for 1, 3, 5, and 7 d, respectively. At the time points indicated above, 90 μL DMEM and 10 μl CCK-8 were added to each well and incubated for an additional 50 min. Then, the media supernatant was removed and the absorbance at 450 nm was measured using a microplate reader (Thermo Fisher Scientific, USA). Wells containing 10% CCK-8 (90 μl DMEM and 10 μl CCK-8) were regarded as blanks. The experiment was repeated three times, with four repeated measures of each experimental value.

For tamoxifen treatment, the experiments were performed according to the previously published data [35]. Briefly, 2 × 10^3^ cells were plated into 96-well plate and culture for 24 h. Then, the cells were washed with 1× PBS, the culture medium was changed with phenol red free DMEM (Gibco, USA) containing 5% charcoal-stripped steroid depleted FBS (Hyclone, USA). Aliquot of gastrin and / or tamoxifen (2 μM) was added to the medium after 24 h culture for 1, 3, 5, and 7 d, respectively. At the time points indicated above, CCK-8 assay was performed to evaluate cell proliferation. The experiment was repeated three times, with four repeated measures of each experimental value.

### IHC

BC and para-BC tissue samples from 50 patients were embedded in paraffin and sliced into 4-μm-thick sections for IHC. After deparaffinization, the sections were placed into a pressure cooker with 10 mM sodium citrate buffer (pH 6.0) at high power for 3 min and then an oven at 100 °C for 15 min, followed by treatment with 3% H_2_O_2_ for 15 min at room temperature. Anti-HER2 (1:200), anti-PR (1:400), anti-ER (1:400), Anti-CCKBR (1:50), anti-p-P65 (1:250), and anti-p-ERK (1:250) primary antibodies were incubated on sections overnight at 4 °C. After being placed at room temperature for 30 min, sections were incubated with a secondary antibody for 15 min. DAB and hematoxylin stained images were obtained on a LEICA microscope (CTR6000) equipped with a digital camera (400X magnification). The positive and negative controls were performed in each IHC experiments simultaneously. The positive controls were stained with the slices that were performed with the same antibody in clinic previously while the negative controls were used PBS instead of the primary antibody in each IHC. In addition, IHCs were performed manually. Three researchers who were blinded to patient prognosis evaluated the slides independently, two of them were pathologists. The criteria for “para BC” regions of the slide were that we collected the tissues distance from cancer 3 cm, which do not include fat tissue. The criteria for the cutoff between positive and negative staining as follows: less than 10% of expression was considered to be “loss” (−), and more than 10% of expression was designated (+).

### Transfection and small interfering RNA (siRNA) treatment

The siRNAs targeting CCKBR and negative control siRNAs were obtained from Shanghai Gene Pharma (Shanghai, China). The siRNA transfection was performed using Lipofectamine 3000 (Thermo Fisher Scientific, USA) according to the manufacturer’s instructions. Briefly, 2 × 10^5^ cells plated in the 6-well plate were transfected. The siRNAs (20 nM, 8 μl) were incubated in OMEM (250 μl) for 5 min while Lipofectamine 3000 reagent (5 μl) was diluted into OMEM (250 μl) gently for 5 min either. Then the above two dilutions were mixed and Incubated for 20 min at room temperature. Lastly DNA-lipid complex was added to cells.

- **siRNAs sequence:**
  - Sense: 5′- GAGCUGGCCAUUAGAAUCATT -3′,
  - Antisense: 5′- UGAUUCUAAUGGCCAGCUCTT -3′.
- **NC sequence:**
  - Sense: 5′- UUCUCCGAACGUGUCACGUTT -3′,
  - Antisense: 5′-ACGUGACACGUUCGGAGAATT -3′.

### Statistical analysis

Statistical analysis was performed using the SPSS v. 21. A chi-square test was used to assess associations between categorical data. A Student’s *t*-test was used for continuous variables using GraphPad Prism v. 6.0. All results are presented as means ± standard error of the means. A *P* < 0.05 was considered significant for all statistical tests.

## Results

### Low gastrin/CCKBR/ERK/P65 level was associated with poor prognosis of ER^+^ BC subtype

**Fig. 1:**
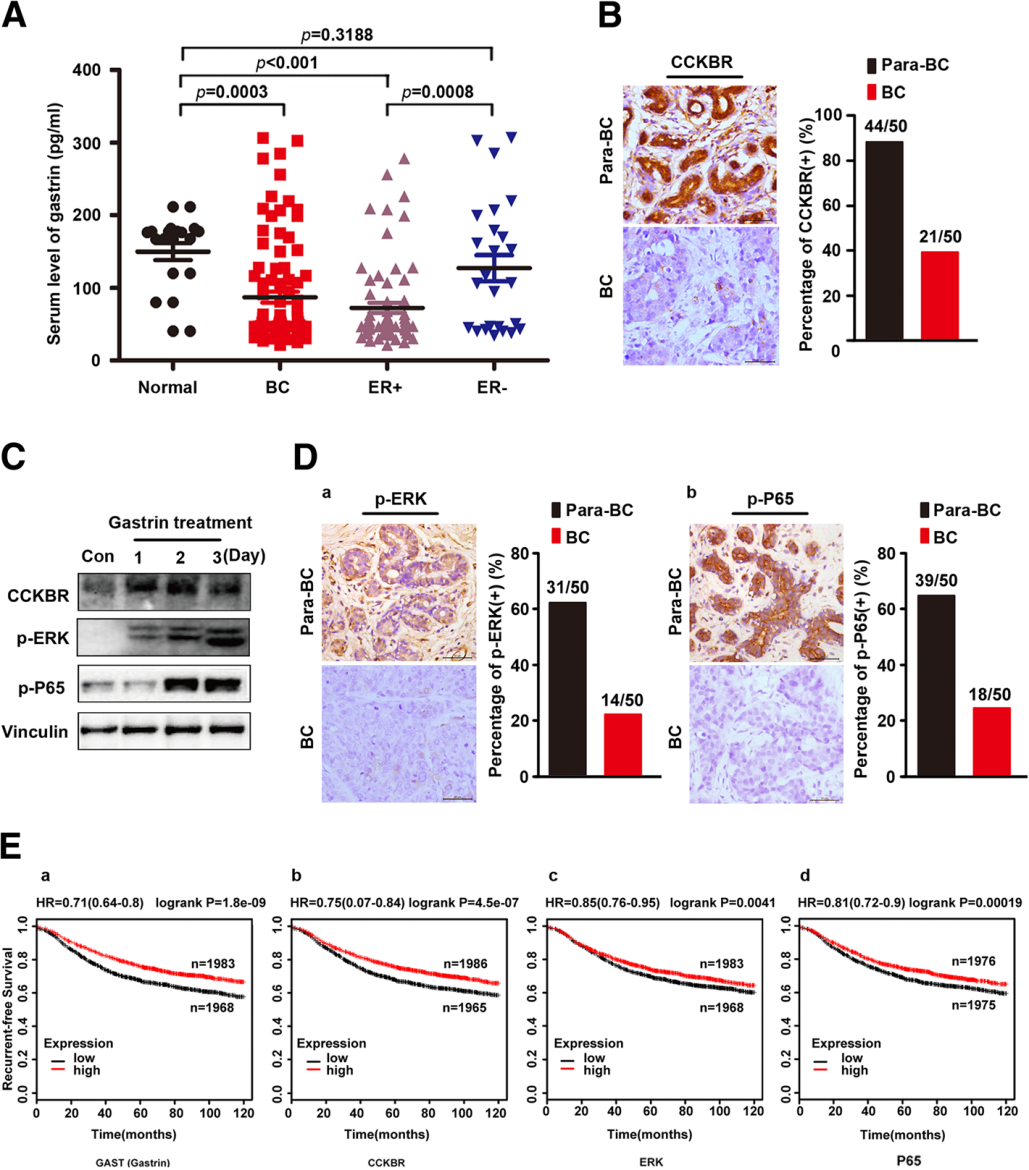
Low gastrin/CCKBR/ERK/P65 level was associated with poor prognosis of ER^+^ BC subtype. **a** Serum levels of gastrin in total, ER^+^ and ER^−^ BC patients and controls (total patients, *N* = 93; ER^+^, *N* = 73; ER^−^, *N* = 20; controls, *N* = 20, *P < 0.05). **b** Expression of CCKBR in 50 paired primary BC and para-BC tissue samples of ER^+^ subtype. A representative IHC of CCKBR in 50 paired primary BC and para-BC tissue samples of ER^+^ subtype (left). Percentage of cases expressing CCKBR in 50 paired primary BC and para-BC tissue samples of ER^+^ subtype (right). **c** CCKBR was increased in MCF-7 cells treated with gastrin as well as p-ERK and p-P65. **d** Expression of p-ERK and p-P65 in 50 paired primary BC and para-BC tissue samples of ER^+^ subtype. (a) A representative IHC of p-ERK in 50 paired primary and para-BC tissue samples of ER^+^ subtype (left). Percentage of cases expressing p-ERK in 50 paired primary BC and para-BC tissue samples of ER^+^ subtype (right). (b) A representative of IHC of p-P65 in 50 paired primary and para-BC tissue samples of ER^+^ subtype (left). Percentage of cases expressing p-P65 in 50 paired primary BC and para-BC tissue samples of ER^+^ subtype (right). **e** Kaplan-Meier survival analysis for the relationship between survival time and different signatures (GAST, CCKBR, ERK, P65) in breast cancer was performed by using the online tool (http://kmplot.com/analysis/)

In order to explore the role of gastrin in BC, we first measured the serum level of gastrin in 93 BC patients and 20 control subjects. The results showed that gastrin levels were significantly reduced in the majority of BC patients compared to normal controls (Fig. 1A). Importantly, low gastrin level was correlated to clinicopathological characters involving ER subtype and tumor size (Table 1). Particularly, serum gastrin level in ER^+^ BC patients was further decreased indicating a correlation between gastrin and this special subtype of BC. Given that CCKBR served as the receptor of gastrin, it was also determined in BC patients through IHC, and the results showed CCKBR expression was also markedly reduced in this subtype of BC (Fig. 1B), which was increased in MCF-7 cells treated with gastrin (Fig. 1C). According to previous reports that ERK could be activated by downstream CCKBR signal, p-ERK was stimulated in gastrin-treated BC cells as well as p-P65 which was phosphorylated by p-ERK (Fig. 1C). Consistently, p-ERK and p-P65 were decreased in ER^+^ BC subtypes compared to adjacent normal tissues (Fig. 1D). Furthermore, the association of four gene expressions with relapse free survival (RFS) was performed using the KM Plotter Online Tool (http://www.kmplot.com) and the results suggested that the expression level of gastrin/CCKBR/ERK/P65 was found to be correlated with better RFS in BC (Fig. 1E).

**Table 1:** Association between gastrin levels and clinicopathological variables of 93 breast cancer cases

Clinicopathological parameters	N^a^	Gastrin levels (*p*g/ml)		χ^2^	*P* Value
		<149^b^	≥149		
All	93	75	18		
Age(year)				0.155	0.694
<45	40	33	7		
≥ 45	53	42	11		
Grade(WHO)				2.538	0.281
I	11	8	3		
II	70	59	11		
III	12	8	4		
Histopathological type				14.530	0.000*
ER^+^	69	62	7		
ER^−^	24	13	11		
Tumor size (cm)				11.627	0.001*
<2	16	8	8		
≥ 2	77	67	10		
Local lymph node metastasis				0.000	0.986
yes	36	29	7		
no	57	46	11		

^a^Number of cases in each group

^b^The mean value of serum gastrin level of normal persons

*Statistically significant (*P* < 0.05)

**Fig. 2:**
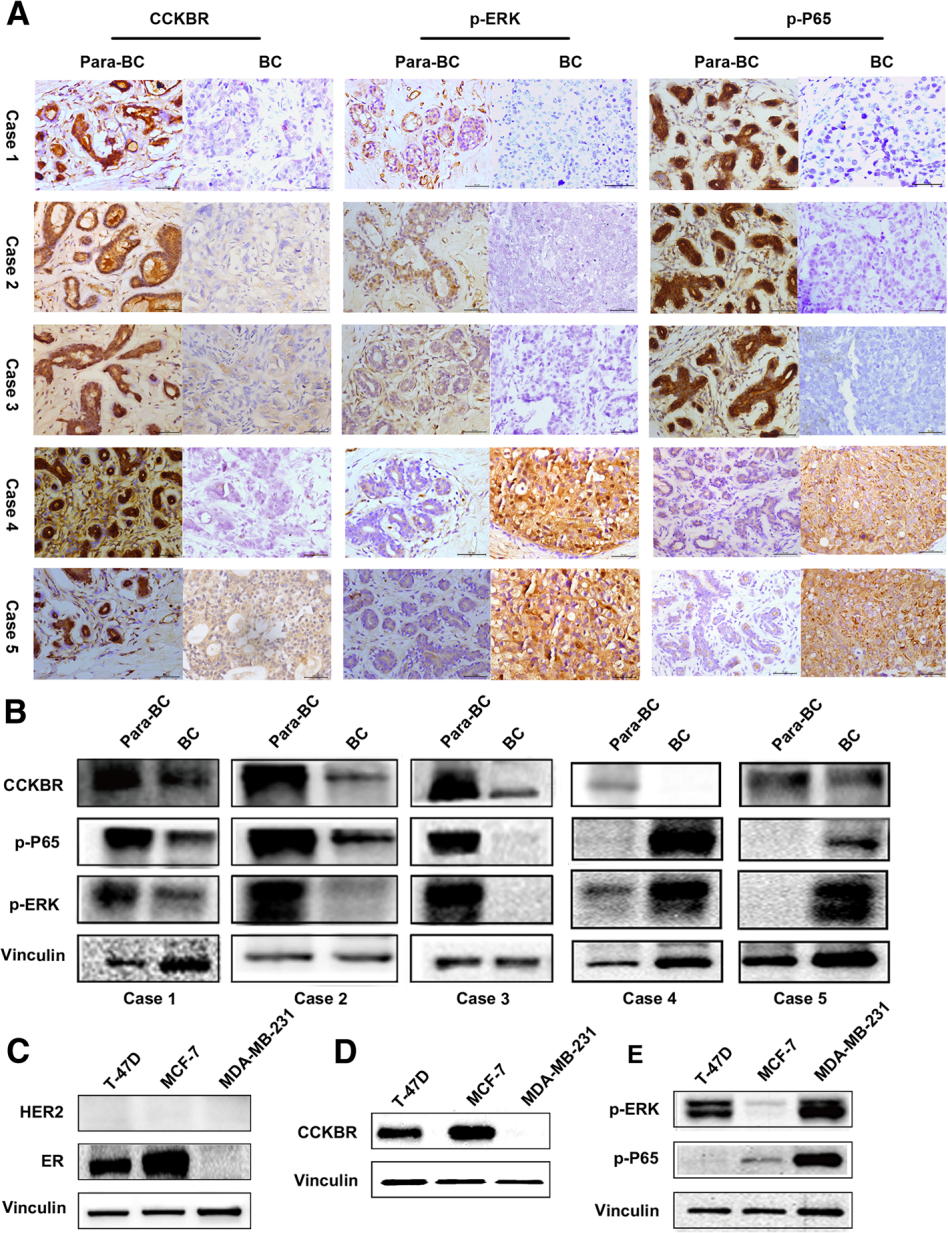
Expression of CCKBR/p-ERK/p-P65 in ER^+^ and ER^−^BC tissues. **a** Expression of CCKBR, p-ERK and p-P65 in 5 paired primary BC and para-BC tissues of different subtypes detected by IHC. Case1:HER2^−^ER^+^PR^+^ Case2:HER2^−^ER^+^PR^+^ Case3:HER2^−^ER^+^PR^+^ Case4:HER2^−^ER^−^PR^−^(TNBC) Case5: HER2^+^ER^−^PR^−^ Scale bar: 50 μm. **b** Expression of CCKBR, p-ERK and p-P65 in 5 paired primary BC and para-BC tissues of different subtypes detected by Western blot. (**c**-**e**) Expression of CCKBR, p-ERK and p-P65 in MCF-7(ER^+^), T-47D(ER^+^) and MDA-MB-231(ER^−^) cells detected by Western blot

To further confirm the association of CCKBR/ERK/P65 and ER positive BC, the levels of CCKBR, p-ERK and p-P65 in fresh tumor and corresponding adjacent normal BC tissues (*N* = 5) were determined by western blot and IHC (ER, PR and HER2 status was determined in Additional file 2: Figure S1). The results indicated that expressions of CCKBR, p-ERK and p-P65 were all decreased in these examples of ER^+^ subtype, but not in ER^−^ or TNBC BC subtype (Fig. 2A and B). Of note, expression of ERK/P65 was activated in TNBC and ER^−^ BC with or without reduction of CCKBR, suggesting ERK/P65 might be under the regulation of other signaling pathways in these two molecular subtypes of BC (Fig. 2). These results were confirmed by experiments in MCF-7 (ER^+^), T-47D (ER^+^), and MDA-MB-231 (ER^−^) BC cell lines. Moreover, expression of p-ERK/p-P65 was decreased in MCF-7 and T-47D versus MDA-MB-231 cells (Fig. 2C-E).

### Gastrin inhibits growth of ER^+^ BC through CCKBR-mediated upregulation of p-ERK/p-P65

**Fig. 3:**
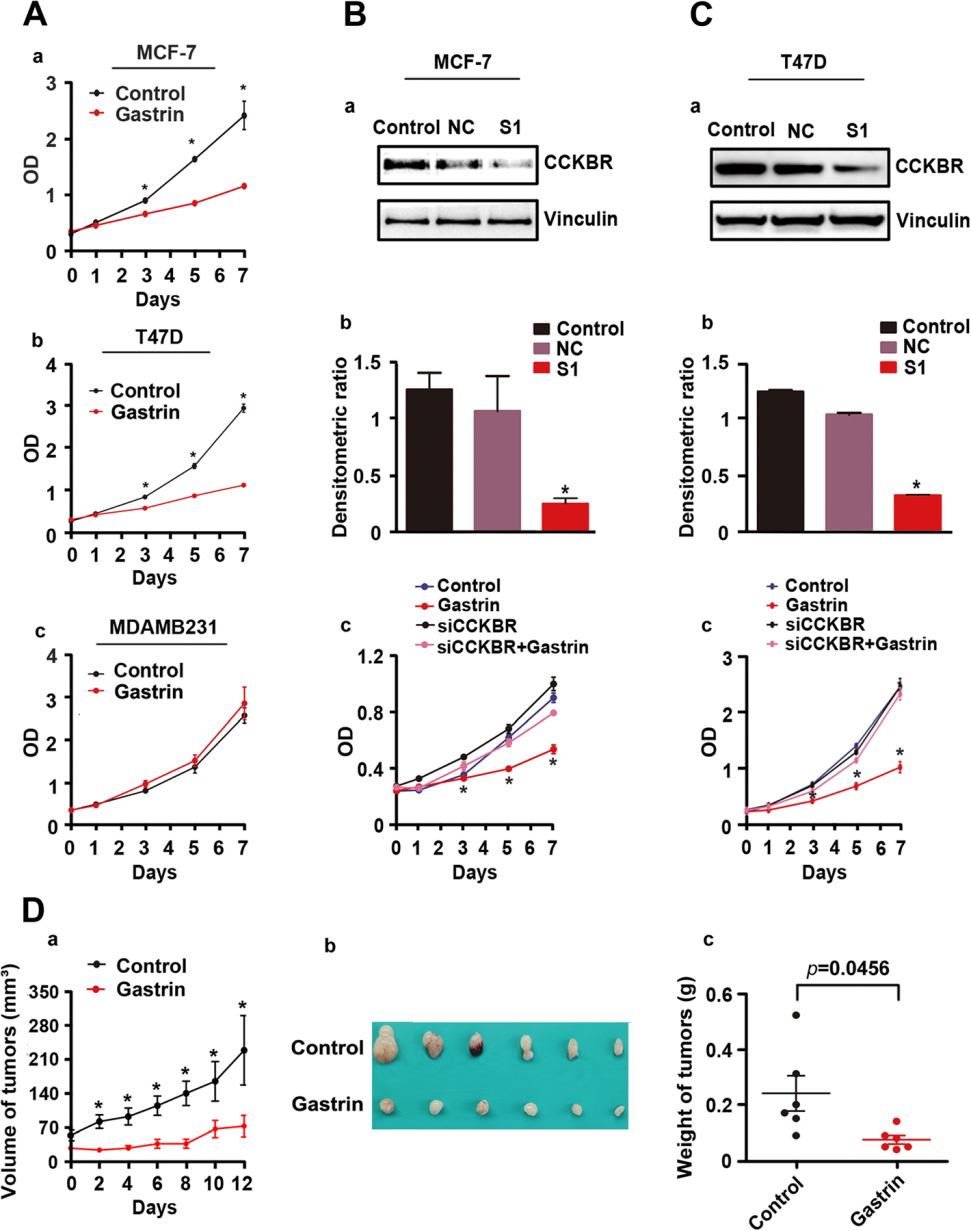
CCKBR mediated suppressive effects of gastrin on ER^+^ BC cells. **a** ER^+^ MCF-7, as well as T-47D and ER^−^ MDA-MB-231 BC cells were treated with gastrin (10^− 7^ M) for 7 d. CCK-8 assay results demonstrated that gastrin inhibited proliferation of MCF-7 (a) and T-47D (b), but not MDA-MB-231 (c) cells. (**b**-**c**) Knockdown of CCKBR by CCKBR-targeted siRNA blocked the effects of gastrin on ER^+^ BC cells. (a) Expression of CCKBR in MCF-7 and T-47D cells transfected with CCKBR-targeted siRNA for 48 h. (b) Gray density analysis demonstrated about two-thirds of CCKBR were downregulated in MCF-7 and T-47D cells (*P < 0.01). (c) CCK-8 assay results demonstrated that knockdown of CCKBR blocked the inhibitory effects of gastrin on MCF-7 and T-47D cells (*P < 0.05). **d** MCF-7 BC tumors grew much slower in animals treated with gastrin. (a) Growth curves of MCF-7 tumors in control and experimental mice (*P < 0.01). (b) The panel shows tumors removed from mice 12-d post-gastrin treatment (control, *N* = 6; gastrin treatment, *N* = 6). (c) Weight of tumors removed from mice 12-d post-gastrin treatment (control, *N* = 6; gastrin treatment, *N* = 6; *P < 0.05)

To explore the role of gastrin in BC, MCF-7, T-47D, and MDA-MB-231 cells were treated with gastrin for 7 days, and the CCK-8 assay was performed at each time point. The results showed that gastrin inhibited growth of MCF-7 and T-47D cells, but not MDA-MB-231 cells (Fig. 3A). To test whether gastrin plays a CCKBR-dependent role, CCKBR was knockdown by the targeted siRNAs (S1, most efficient) in MCF-7 and T-47D cells co-treated with gastrin. As shown in Fig. 3B and C, S1 significantly inhibited expression of CCKBR and gastrin–mediated inhibition on proliferation of BC cells was greatly weakened in these BC cells. In addition, the inhibitory effect of gastrin on ER^+^ BC was further confirmed in mice bearing MCF-7 tumors (Fig. 3D).

**Fig. 4:**
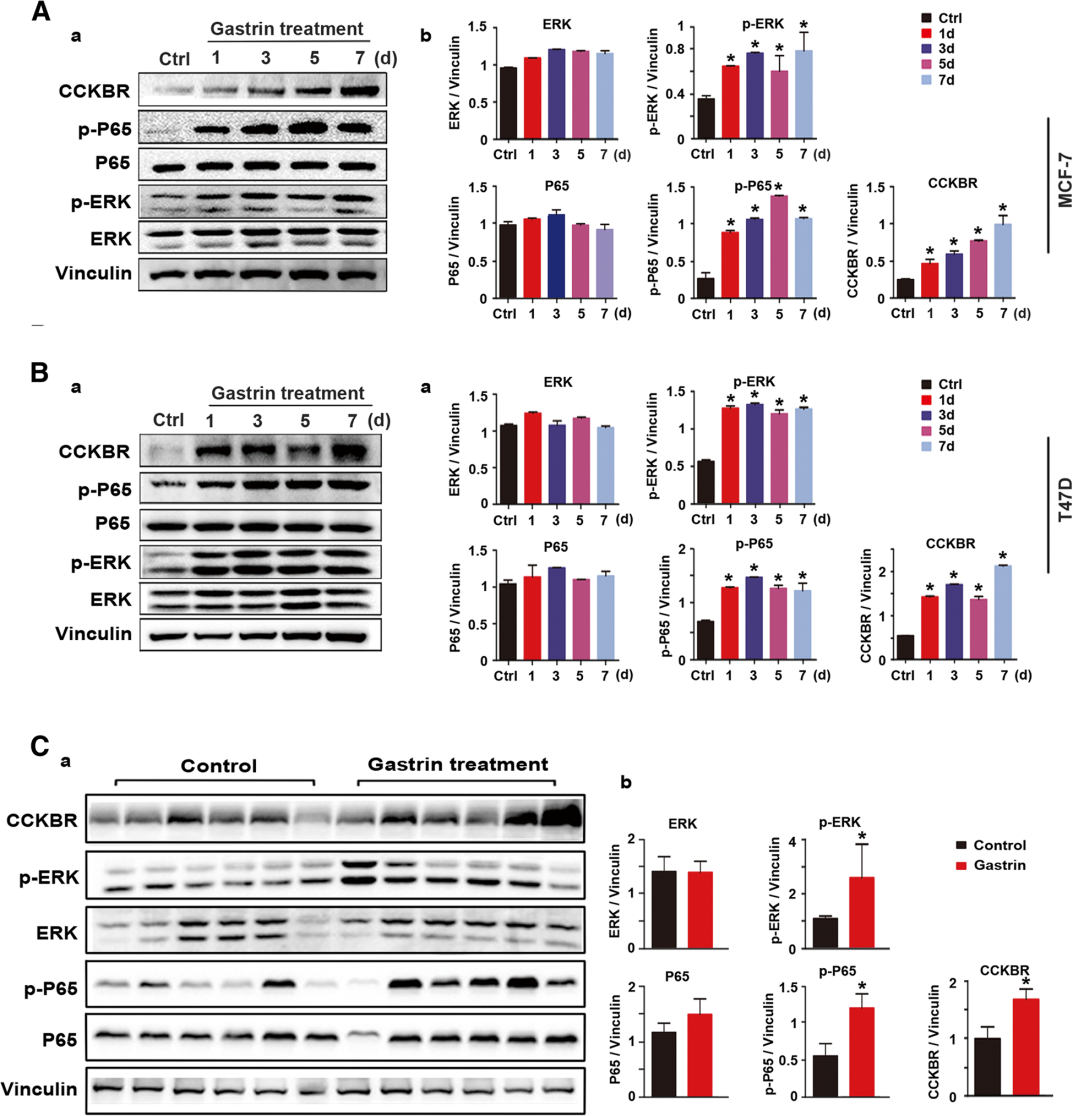
Gastrin inhibited growth of ER^+^ BC through upregulation of CCKBR/p-ERK/p-P65. (**a**-**b**) Left panels, expression of CCKBR, ERK/P65, and p-ERK/p-P65 in ER^+^ MCF-7 and T-47D cells (from Fig. 3A, a and b) detected by Western blot. Right panels, gray density analysis of bands in left panels. **c** Expression of CCKBR, ERK/P65, and p-ERK/p-P65 in tumors shown in Fig. 3C detected by Western blot. Bands in the upper panel were quantified by gray density analysis and shown in the lower panel. **d** Expression of CCKBR, ERK/P65, and p-ERK/p-P65 in tumors shown in Fig. 1C detected by Western blot. Bands in left panel were quantified by gray density analysis and shown in the lower panel

To define the role of gastrin in BC through CCKBR-mediated regulation of p-ERK/p-P65, expression of p-ERK/p-P65 in BC cell lines and mice bearing tumors was detected by Western blot. As show in Fig. 4, gastrin treatment led to up-regulation of CCKBR/p-ERK/p-P65 along with growth inhibition of MCF-7 and T-47D cells (Fig. 3A).

### ERK/P65 regulated the proliferation of ER^+^ BC

**Fig. 5:**
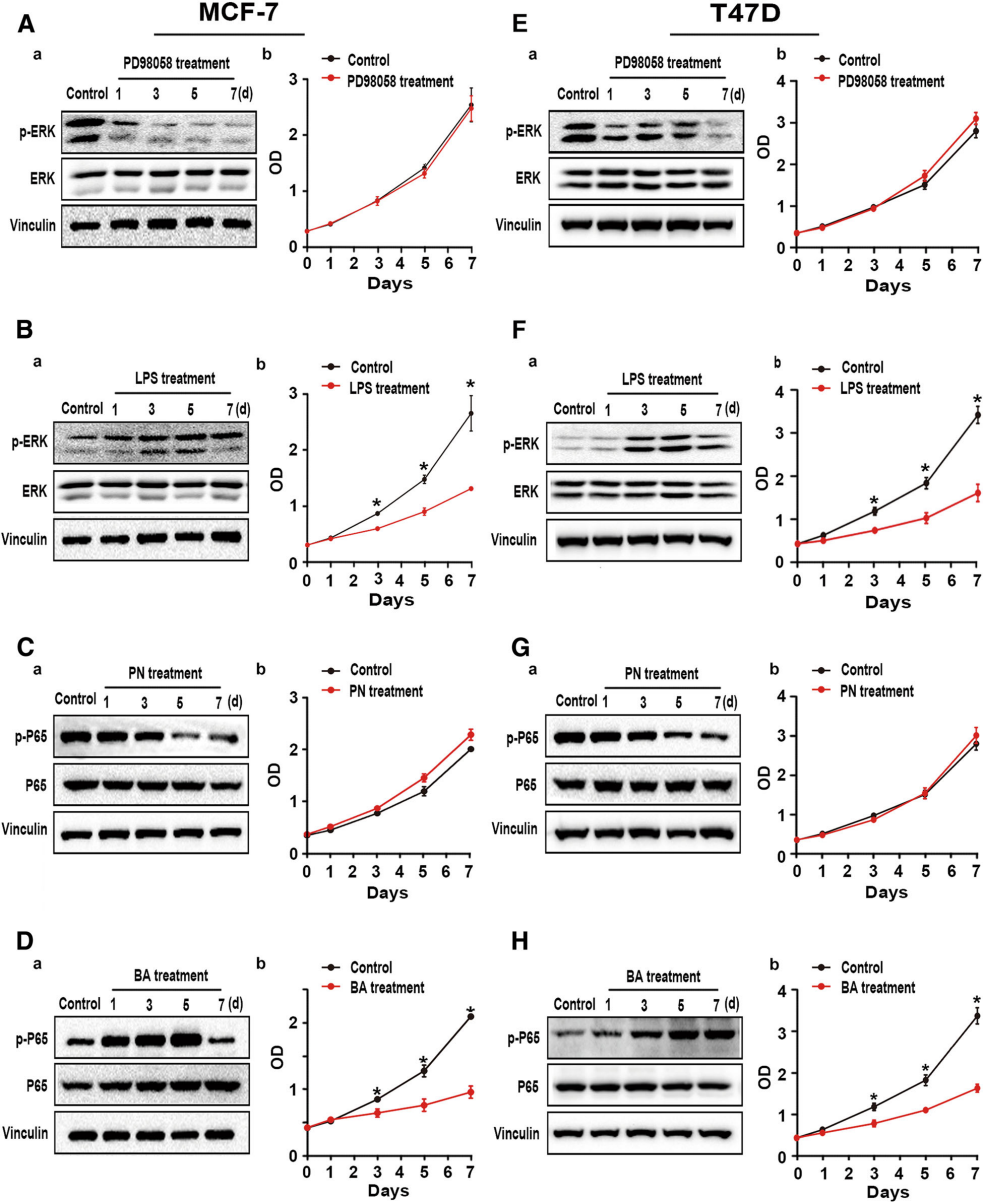
Effects of inhibitors/activators of ERK/P65 on ER^+^ BC cells. (**a**, **b**, **e**, and **f**) MCF-7 and T-47D cells treated with ERK inhibitor PD98059 (10 μM) or lipopolysaccharide (1 μg/ml) as an activator for 7 d were harvested at each time point indicated and subjected to CCK-8 assay (right) and Western blot (left) [*P < 0.05]. (**c**, **d**, **g**, and **h**) MCF-7 and T-47D cells treated with P65 inhibitor parthenolide (10 μg/ml) or betulinic acid (10 μg/ml) as an activator for 7 d were harvested at each time point indicated and subjected to CCK-8 assay (right) and Western blot (left) [*P < 0.05]

To test whether ERK/P65 activator also inhibits growth of ER^+^/CCKBR^−^/ERK^−^/P65^−^ BC cells, MFC-7 and T-47D cells were treated with the activators and the inhibitors of ERK/P65, respectively. As shown in Fig. 5, the ERK/P65 inhibitors significantly inhibited expression of p-ERK/p-P65 without growth inhibition of either cell line (Fig. 5 A, E, C, and G). While the ERK/P65 activators activated the two proteins, they also inhibited proliferation of cells (Fig. 5B, F, D, and H).

### Gastrin and tamoxifen synergistically affect BC suppression

**Fig. 6:**
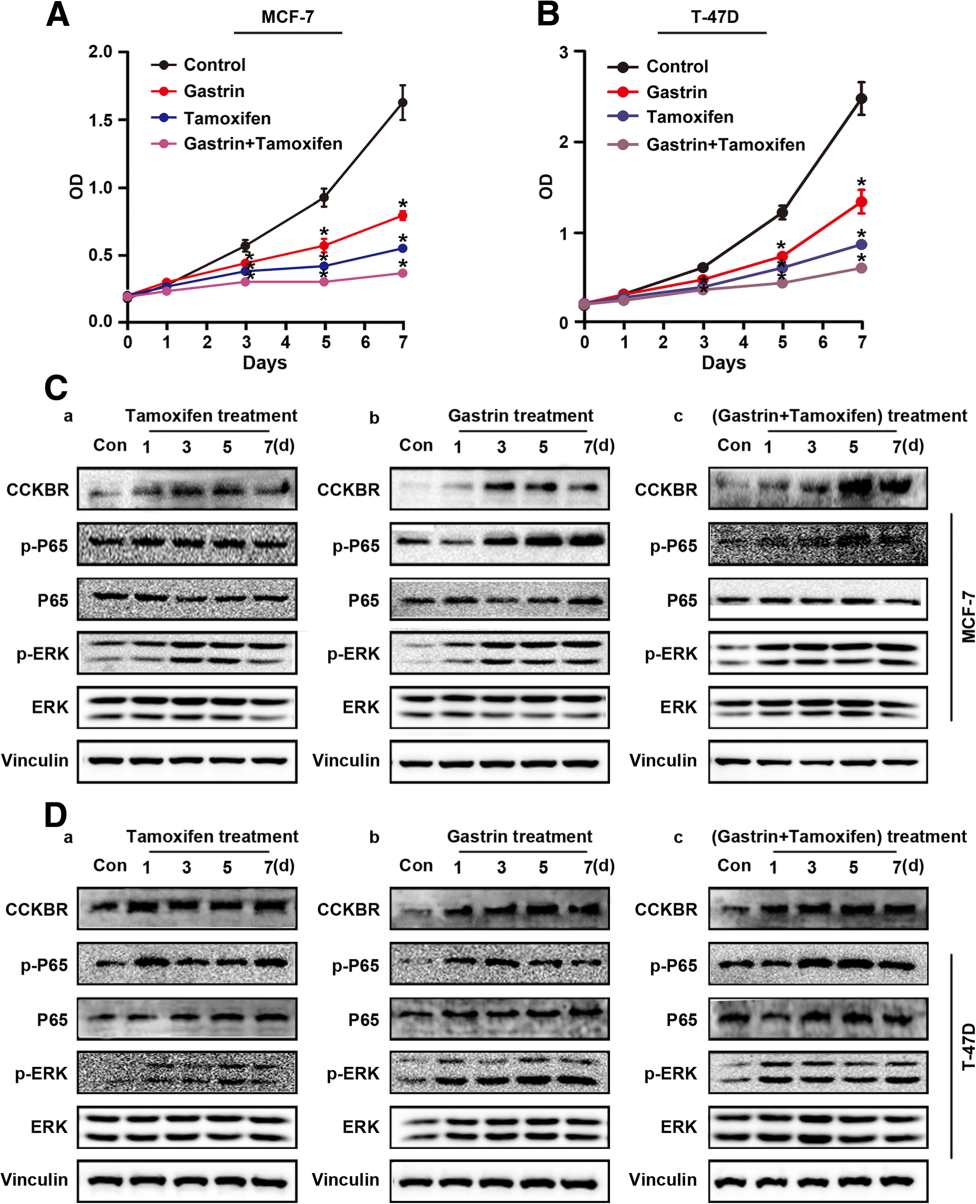
Effect of gastrin and tamoxifen treatment on growth of ER^+^ MCF-7 and T-47D cells. (**a**-**b**) MCF-7 and T-47D cells treated with gastrin (10^− 7^ M), tamoxifen (2 μM), or combination of both for 7 d were harvested at each time point for CCK-8 assay and Western blot. (A) CCK-8 assay of MCF-7 cells; (B) CCK-8 assay of T-47D cells (*P < 0.05). **c** Activation of CCKBR/ERK/P65 signaling in MCF-7 cells treated with tamoxifen (a), gastrin (b), or a combination of both agents (c). **d** Activation of CCKBR/ERK/P65 signaling in T-47D cells treated with tamoxifen (a), gastrin (b), or a combination of both agents (c)

Tamoxifen is currently used for the treatment of ER^+^ BC. It is also approved by the US Food and Drug Administration for the prevention of BC in women at high risk for developing the disease. However, the underlying mechanism is still not fully understood. If the inactivation of CCKBR/ERK/P65 is a key event in BC development, then tamoxifen must affect expression of CCKBR/p-ERK/p-P65 in BC cells. To test this, MCF-7 and T-47D cells were separately treated with tamoxifen or gastrin alone or in combination. The results showed that both agents remarkably inhibited growth of the both BC cell lines (Fig. 6). Notably, combination treatment produced a synergistic inhibitory effect (Fig. 6A and B). As with gastrin treatment, tamoxifen also increased expression of CCKBR/p-ERK/p-P65 in both BC cell lines, and it is likely that tamoxifen and gastrin play a cooperative role in down-regulation of CCKBR/p-ERK/p-P65 (Fig. 6C and D).

## Discussion

The present study showed for the first time that low serum levels of gastrin closely correlate with BC development via ER^+^/CCKBR^−^/p-ERK^−^/p-P65^−^. Importantly, gastrin was indicated to protect the breast gland and inhibit growth of BC via CCKBR-mediated activation of ERK/P65 signaling. The results demonstrated that gastrin and its receptor CCKBR could prevent carcinogenesis within the breast gland, and a low level of gastrin was a risk factor for BC development, especially in ER^+^ BC.

Overexpression of CCKBR described in several types of cancers led to the development of CCKBR-targeted therapeutic agents. However, there has been still no successful clinical trials reported [36, 37]. Recently, it was reported that CCKBR expressed in almost all kinds of cancers, and none of the cancer samples showed the higher CCKBR expression than that in the paired non-cancer samples [37]. Our current results indicated the decreased CCKBR expression in ER^+^ BC (Fig. 2A and B), deficient expression in ER^−^ BC (Fig. 2A and B), and no difference of expression in ER^−^ BC (Fig. 2A and B). Consistent expression of CCKBR, p-ERK and p-P65 were also up-regulated by gastrin which indicated a link between the gastrin/CCKBR/ERK/P65 pathway and ER^+^ BC subtype. Thus, inactivation of ERK/P65 in BC was related to low serum levels of gastrin and down-regulated expression of CCKBR.

ERK, a member of the RAS/RAF/MEK/ERK pathway, was established as a major participant in the regulation of cell growth and differentiation. ERK1/2 forms a central component in the MAPK/ERK cascade and was improperly activated in several types of cancers [38–40]. However, other than lapatinib that was applied to treat HER2^+^/ERK^+^ BC [41–43], there have been few reports of successful ERK1/2 inhibitors for BC suppression. It suggests that ERK1/2 inhibition is invalid for most of BC patients. The functions of ERK1/2 in BC appear to be complex due to several cellular responses and their interaction with different pathways, including key genes in BC (ER and HER2) [44–46].

Our results indicated that ERK was inactivated in most of BC, consistent with ER expression. Similar results were reported by Ahmad et al., who assessed ERK expression by IHC in a large series of BC samples and found that ERK1/2 were associated with a good prognosis, and their expression was mainly related to ER [46]. The current results demonstrated that BC had a CCKBR/p-ERK/p-P65-negative molecular subtype which corresponded to ER^+^, indicating that clinical trials targeting ERK1/2 could have been based on incorrect assumptions due to the complexity of ERK context. It is not inactivation of ERK1/2, but rather their activation suppresses BC growth. Thus, we propose that the therapies of CCKBR/p-ERK/p-P65-negative BC should target the signaling to restore ERK/P65 activity rather than to inhibit it.

Once activated, further phosphorylation of ERK1/2 can activate transcription factors, including P65, a component of the NF-κB family [47, 48]. We found that the activity of P65 in BC tissues was consistent with that of ERK1/2; thus, ER^+^ BC showed low/absent activity of P65, while it was detectable in ER^−^/ERK^+^ BC cells. Another consistent report presented that ER and P65 were generally thought to repress the activities by each other, and activation of P65 in BC was common and typically associated with the loss of ER, which might be mediated by HER2 overexpression [46, 49]. We also found that both tamoxifen and gastrin inhibited the growth of ER^+^ BC via up-regulating CCKBR and p-ERK/p-P65. Moreover, these two agents played the cooperative roles in BC cell suppression.

Activation of NF-κB, as well as ER/NF-κB crosstalk, is significantly associated with aggressive disease and poor patient outcome in women with ER positive breast cancer [50–52]. However, it is not fully understood whether NF-κB is a driver or a consequence of aggressive ER positive disease. In this study, the reduced p-P65 expression was detected in the patients with ER^+^ BC. Further, we also found P65 phosphorylation was activated by gastrin stimulation in ER positive BC cell lines. The similar results were also described that NF-κB could work cooperatively with ER to inhibit the proliferation of ER positive BC cells [53, 54]. Despite a considerable body of evidence supporting the role for the NF-κB pathway in aggressive ER^+^ breast tumors, the precise mechanism also need to be further investigated.

In the current study, we firstly explored the association between serum level of gastrin and ER positive breast cancer which was never documented before. Second, gastrin inhibited the proliferation of ER positive BC through activating ERK/P65 cascades by binding to its receptor CCKBR. Regarding to the inhibitory role of gastrin on ER positive BC, the combinational effect of gastrin and tamoxifen was also determined on ER positive BC and discussed in the last. Thus, our results indicated that gastrin might have a promising potent on ER positive BC treatment. The results in the present study offered new insights into the molecular mechanisms of the conjunction effects of gastrin and tamoxifen on BC treatment. In particular, it was found that gastrin had the potential in the treatment of ER^+^/CCKBR^−^/p-ERK^−^/p-P65^−^ BC.

## Conclusions

We concluded that low serum gastrin is related to increased risk of ER^+^ BC development. The results also established that CCKBR/ERK/P65 signaling function is generally tumor suppressive in ER^+^ BC, indicating therapies should focus on restoring, not inhibiting, CCKBR/ERK/P65 pathway activity.

## Additional files

Additional file 1: **Table S2.** Clinical information of 93 BC patients. Abbreviations: BC, breast cancer; GB, gastric biopsy; F, female; +, lymphatic metastasis present; −, no lymphatic metastasis present; Y/N (GB column), examined/not examined; Y/N (stomach illness without GB column), those with/without stomach illness who did not undergo GB; IDC, invasive ductal cancer; DCIS, ductal cancer in situ; WHO, World Health Organization. (DOCX 36 kb)

Additional file 2: **Figure S1.** Expression of ER, PR, HER2 in 5 primary BC samples. Three primary BC samples were clinically defined as ER^+^/PR^+^/HER2^−^ by IHC. Two primary BC samples were clinically defined as TNBC and ER^−^/PR^−^/HER2^+^ by IHC. Case1:HER2^−^ER^+^PR^+^ Case2:HER2^−^ER^+^PR^+^ Case3:HER2^−^ER^+^PR^+^ Case4:HER2^−^ER^−^PR^−^(TNBC) Case5: HER2^+^ER^−^PR^−^ Scale bar: 50 μm. (TIF 3702 kb)

## Abbreviations

-: no lymphatic metastasis present
+: lymphatic metastasis present
BA: betulinic acid
BC: breast cancer
CCK-8: cell counting kit-8
CCKBR: Cholecystokinin B receptor
DCIS: ductal cancer in situ
ELISA: enzyme-linked immunosorbent assay
ER: estrogen receptor
ERK: extracellular regulated protein kinases
F: female
GB: gastric biopsy
HER2: human epidermal growth factor receptor 2
IDC: invasive ductal cancer
IHC: immunohistochemistry
LPS: lipopolysaccharides
PN: parthenolide
PR: progesterone receptor
TNBC: triple-negative breast cancer
WHO: World Health Organization
Y/N (GB column): examined/not examined
Y/N (stomach illness without GB column): those with/without stomach illness who did not undergo GB
