# Supplementary material for: Low serum gastrin associated with ER+ breast cancer development via inactivation of CCKBR/ERK/P65 signaling
Source: BMC Cancer. 2018 Aug 16;18:824. doi: 10.1186/s12885-018-4717-7 (PMC6097285; doi:10.1186/s12885-018-4717-7)
Supplement: Supplementary file 1 — Table S2. Clinical information of 93 BC patients. Abbreviations: BC, breast cancer; GB, gastric biopsy; F, female; +, lymphatic metastasis present; −, no lymphatic metastasis present; Y/N (GB column), examined/not examined; Y/N (stomach illness without GB column), those with/without stomach illness who did not undergo GB; IDC, invasive ductal cancer; DCIS, ductal cancer in situ; WHO, World Health Organization. (DOCX 36 kb) [file 12885_2018_4717_MOESM1_ESM.docx]

|  | **Table S2. Clinical information for breast cancer patients (*N*=93)** | | | | | | | |  |
| --- | --- | --- | --- | --- | --- | --- | --- | --- | --- |
| **Patient**  **number** | **Size**  **(cm)** | **Lymphatic metastasis** | **Grade**  **(WHO)** | **GB^a^** | **Stomach illness without GB** | **Serum level of gastrin (*p*g/mL)** | **Molecular**  **signature** | **Type of**  **tissue** | |
| 1 | 2*3 | - | II | Y |  | 21.36 | HER2(-) ER(+) PR(+) | IDC**^b^**/DCIS**^c^** | |
| 2 | 5.5*1.5 | + | II | Y |  | 25.28 | HER2(-) ER(+) PR(+) | DCIS | |
| 3 | 4*3 | + | II | Y |  | 27.18 | HER2(-) ER(+) PR(+) | IDC | |
| 4 | 2*3 | - | II | Y |  | 29.02 | HER2(-) ER(+) PR(+) | IDC/DCIS | |
| 5 | 2*2 | - | II | N | Y | 30.33 | HER2(-) ER(+) PR(+) | IDC | |
| 6 | 2*1.5 | - | II | Y |  | 30.47 | HER2(-) ER(+) PR(-) | IDC | |
| 7 | 1.5*1.5 | - | II | N | N | 30.50 | HER2(-) ER(+) PR(-) | IDC | |
| 8 | 2.8*2.8 | - | II | N | N | 31.71 | HER2(-)ER(+) PR(+) | IDC | |
| 9 | 2.6*1.9 | - | II | N | N | 34.29 | HER2(+)ER(-) PR(+) | IDC/DCIS | |
| 10 | 3*2 | - | II | Y |  | 35.10 | HER2(-) ER(+) PR(+) | IDC/DCIS | |
| 11 | 2*2 | - | II | N | Y | 35.79 | HER2(-) ER(+) PR(+) | IDC | |
| 12 | 2.6*0.7 | - | II | N | Y | 35.84 | HER2(-) ER(+) PR(-) | IDC | |
| 13 | 2.4*0.9 | - | II | N | Y | 37.00 | HER2(-) ER(+) PR(-) | IDC | |
| 14 | 6*4 | - | III | Y |  | 37.53 | HER2(-) ER(+) PR(-) | IDC | |
| 15 | 2*3 | - | II | Y |  | 37.90 | HER2(+) ER(-) PR(-) | IDC/DCIS | |
| 16 | 4*5 | - | II | Y |  | 38.01 | HER2(-)ER(+) PR(+) | IDC | |
| 17 | 2*3 | + | II | N | Y | 38.14 | HER2(-) ER(+) PR(+) | IDC/DCIS | |
| 18 | 3*4 | - | II | Y |  | 38.24 | HER2(-) ER(+) PR(+) | IDC | |
| 19 | 2*2 | - | II | N | Y | 39.32 | HER2(-)ER(+) PR(-) | IDC | |
| 20 | 3*4 | + | II | Y |  | 40.21 | HER2(+)ER(-) PR(+) | IDC/DCIS | |
| 21 | 3*4 | + | II | Y |  | 42.00 | HER2(-) ER(+) PR(+) | IDC | |
| 22 | 2*2 | - | II | Y |  | 42.45 | HER2(-) ER(+) PR(+) | IDC/DCIS | |
| 23 | 1.5*1.5 | - | II | N | N | 42.62 | HER2(+)ER(-) PR(+) | IDC | |
| 24 | 2*3 | - | II | Y |  | 43.17 | HER2(+)ER(-) PR(+) | IDC | |
| 25 | 2*2 | + | II | Y |  | 43.58 | HER2(-) ER(+) PR(+) | IDC | |
| 26 | 2*2 | - | II | Y |  | 43.63 | HER2(-) ER(+) PR(+) | IDC/DCIS | |
| 27 | 3*4 | + | II | Y |  | 45.03 | HER2(-) ER(+) PR(+) | IDC | |
| 28 | 2*3 | + | II | Y |  | 45.25 | HER2(-) ER(+) PR(+) | IDC | |
| 29 | 2*2 | + | II | N | N | 45.25 | HER2(+)ER(-) PR(+) | IDC | |
| 30 | 2.9*1.5 | + | II | N | Y | 45.65 | HER2(-) ER(+) PR(+) | IDC | |
| 31 | 1*2 | - | II | N | Y | 46.32 | HER2(-) ER(+) PR(+) | IDC | |
| 32 | 1.2*0.7 | - | I | N | Y | 46.52 | HER2(+) ER(-) PR(-) | IDC | |
| 33 | 3*3 | - | III | Y |  | 46.62 | HER2(-) ER(+) PR(-) | IDC | |
| 34 | 2.5*2 | + | II | N | Y | 46.68 | HER2(-) ER(+) PR(-) | IDC/DCIS | |
| 35 | 4*2.2 | + | III | Y |  | 46.78 | HER2(-) ER(+) PR(-) | IDC | |
| 36 | 2*2 | + | II | N | N | 46.87 | HER2(+) ER(-) PR(-) | IDC | |
| 37 | 3*3 | + | II | Y |  | 46.89 | HER2(-) ER(+) PR(+) | IDC | |
| 38 | 3.2*1.5 | + | II | N | Y | 46.98 | HER2(-) ER(+) PR(-) | IDC/DCIS | |
| 39 | 4*3 | - | II | \| N \| \| --- \| | Y | 47.01 | HER2(-) ER(+) PR(+) | IDC | |
| 40 | 2.6*1.9 | - | III | Y |  | 47.10 | HER2(-) ER(+) PR(+) | IDC/DCIS | |
| 41 | 2*1 | - | I | N | N | 47.15 | HER2(-) ER(+) PR(+) | IDC | |
| 42 | 6*5 | - | II | Y |  | 47.35 | HER2(-) ER(+) PR(+) | IDC/DCIS | |
| 43 | 4*4 | - | I | Y |  | 47.45 | HER2(-) ER(+) PR(+) | IDC | |
| 44 | 2*2 | + | II | N | N | 47.84 | HER2(-) ER(+) PR(+) | IDC | |
| 45 | 2.1*1.1 | - | II | N | Y | 48.67 | HER2(-)ER(+) PR(-) | IDC | |
| 46 | 1.7*0.9 | + | II | N | Y | 50.54 | HER2(-)ER(-) PR(-) | IDC | |
| 47 | 6*6 | + | III | Y |  | 50.54 | HER2(-) ER(+) PR(+) | IDC | |
| 48 | 2.5*1.5 | + | III | Y |  | 52.12 | HER2(-) ER(+) PR(+) | IDC | |
| 49 | 4*2 | + | II | Y |  | 52.18 | HER2(-) ER(+) PR(+) | IDC | |
| 50 | 6*1.7 | - | III | N | N | 52.65 | HER2(-) ER(+) PR(+) | IDC | |
| 51 | 6*6 | + | II | N | Y | 52.75 | HER2(-) ER(+) PR(+) | IDC/DCIS | |
| 52 | 2.5*1 | - | II | Y |  | 54.18 | HER2(-) ER(+) PR(+) | IDC | |
| 53 | 1.5*2.7 | - | II | Y |  | 54.29 | HER2(-) ER(+) PR(-) | IDC | |
| 54 | 2*2 | + | II | Y |  | 54.30 | HER2(-) ER(+) PR(+) | IDC/DCIS | |
| 55 | 2*3 | - | II | N | N | 54.45 | HER2(-)ER(+) PR(+) | IDC/DCIS | |
| 56 | 2*3 | - | III | N | Y | 58.47 | HER2(-) ER(+) PR(+) | IDC/DCIS | |
| 57 | 1*2 | + | II | N | Y | 58.90 | HER2(-) ER(+) PR(-) | IDC | |
| 58 | 4*3 | + | II | Y |  | 59.88 | HER2(-) ER(+) PR(+) | IDC | |
| 59 | 6*4 | + | II | N | Y | 62.81 | HER2(-) ER(+) PR(+) | IDC/DCIS | |
| 60 | 2*1 | - | I | N | Y | 63.90 | HER2(-) ER(+) PR(+) | IDC | |
| 61 | 1*1 | - | I | Y |  | 68.12 | HER2(-)ER(+) PR(+) | IDC | |
| 62 | 6*1.7 | + | II | Y |  | 81.21 | HER2(-) ER(+) PR(+) | IDC/DCIS | |
| 63 | 1*1 | - | II | Y |  | 82.86 | HER2(-) ER(+) PR(+) | IDC | |
| 64 | 6*6 | - | II | N | Y | 91.51 | HER2(-) ER(+) PR(+) | IDC | |
| 65 | 3*4 | - | II | N | N | 94.99 | HER2(+) ER(-) PR(-) | DCIS | |
| 66 | 1.6*1.5 | - | II | N | N | 106.20 | HER2(+) ER(-) PR(-) | IDC | |
| 67 | 4*3 | - | II | Y |  | 106.78 | HER2(+) ER(-) PR(-) | IDC | |
| 68 | 3*4 | - | II | Y |  | 107.45 | HER2(-) ER(+) PR(+) | IDC | |
| 69 | 2*5 | - | II | N | N | 111.07 | HER2(-)ER(+) PR(+) | IDC | |
| 70 | 2*2 | - | I | Y |  | 111.50 | HER2(-) ER(+) PR(+) | IDC | |
| 71 | 2.6*1.9 | - | II | N | N | 116.78 | HER2(+) ER(-) PR(-) | IDC | |
| 72 | 7*5 | + | II | Y |  | 117.32 | HER2(-) ER(+) PR(+) | IDC | |
| 73 | 2*2 | - | I | N | Y | 126.98 | HER2(-) ER(+) PR(-) | IDC | |
| 74 | 1.1*0.8 | + | I | N | Y | 127.98 | HER2(-) ER(+) PR(-) | IDC | |
| 75 | 2*3 | + | II | Y |  | 128.09 | HER2(-) ER(+) PR(-) | IDC/DCIS | |
| 76 | 2.5*1.5 | + | III | N | Y | 150.11 | HER2(-)ER(-) PR(-) | IDC | |
| 77 | 7*7 | + | III | Y |  | 152.18 | HER2(+) ER(-) PR(-) | IDC/DCIS | |
| 78 | 2*4 | - | III | N | N | 161.13 | HER2(+) ER(-) PR(-) | IDC | |
| 79 | 4*5 | - | III | N | N | 169.38 | HER2(+) ER(-) PR(-) | IDC | |
| 80 | 1*2 | - | I | Y |  | 175.05 | HER2(-) ER(+) PR(+) | IDC/DCIS | |
| 81 | 1.5*1 | - | II | N | N | 178.90 | HER2(+) ER(-) PR(-) | IDC | |
| 82 | 2*2 | + | II | Y |  | 199.09 | HER2(-) ER(+) PR(+) | IDC | |
| 83 | 1*1 | - | II | N | N | 199.12 | HER2(-)ER(-) PR(-) | IDC | |
| 84 | 2*1 | + | II | N | Y | 206.69 | HER2(+) ER(-) PR(-) | IDC | |
| 85 | 2*3 | - | II | N | Y | 207.82 | HER2(-) ER(+) PR(-) | IDC | |
| 86 | 2.5*0.8 | - | I | N | Y | 209.00 | HER2(-) ER(+) PR(-) | IDC/DCIS | |
| 87 | 2.4*0.9 | - | II | N | N | 219.38 | HER2(-)ER(-) PR(-) | IDC | |
| 88 | 1.5*1.5 | + | II | N | N | 226.18 | HER2(-)ER(+) PR(+) | IDC | |
| 89 | 8*7 | - | I | Y |  | 256.19 | HER2(-) ER(+) PR(+) | IDC | |
| 90 | 2*2 | - | II | Y |  | 278.44 | HER2(-) ER(+) PR(+) | IDC | |
| 91 | 2*3 | + | II | N | N | 285.16 | HER2(+)ER(-) PR(+) | IDC | |
| 92 | 2*3 | + | II | N | N | 302.71 | HER2(+) ER(-) PR(-) | IDC | |
| 93 | 1*1 | - | II | Y |  | 306.78 | HER2(+) ER(-) PR(-) | IDC/DCIS | |
| **^a^** gastric biopsy | | | | | | | | |  |
| **^b^** infitrating ductal carcinoma | | | | | | | | |  |
| **^c^** ductal carcinoma in situ | | | | | | | | |  |
