# Supplementary figures and images for: Low serum gastrin associated with ER+ breast cancer development via inactivation of CCKBR/ERK/P65 signaling
Source: BMC Cancer. 2018 Aug 16;18:824. doi: 10.1186/s12885-018-4717-7 (PMC6097285; doi:10.1186/s12885-018-4717-7)

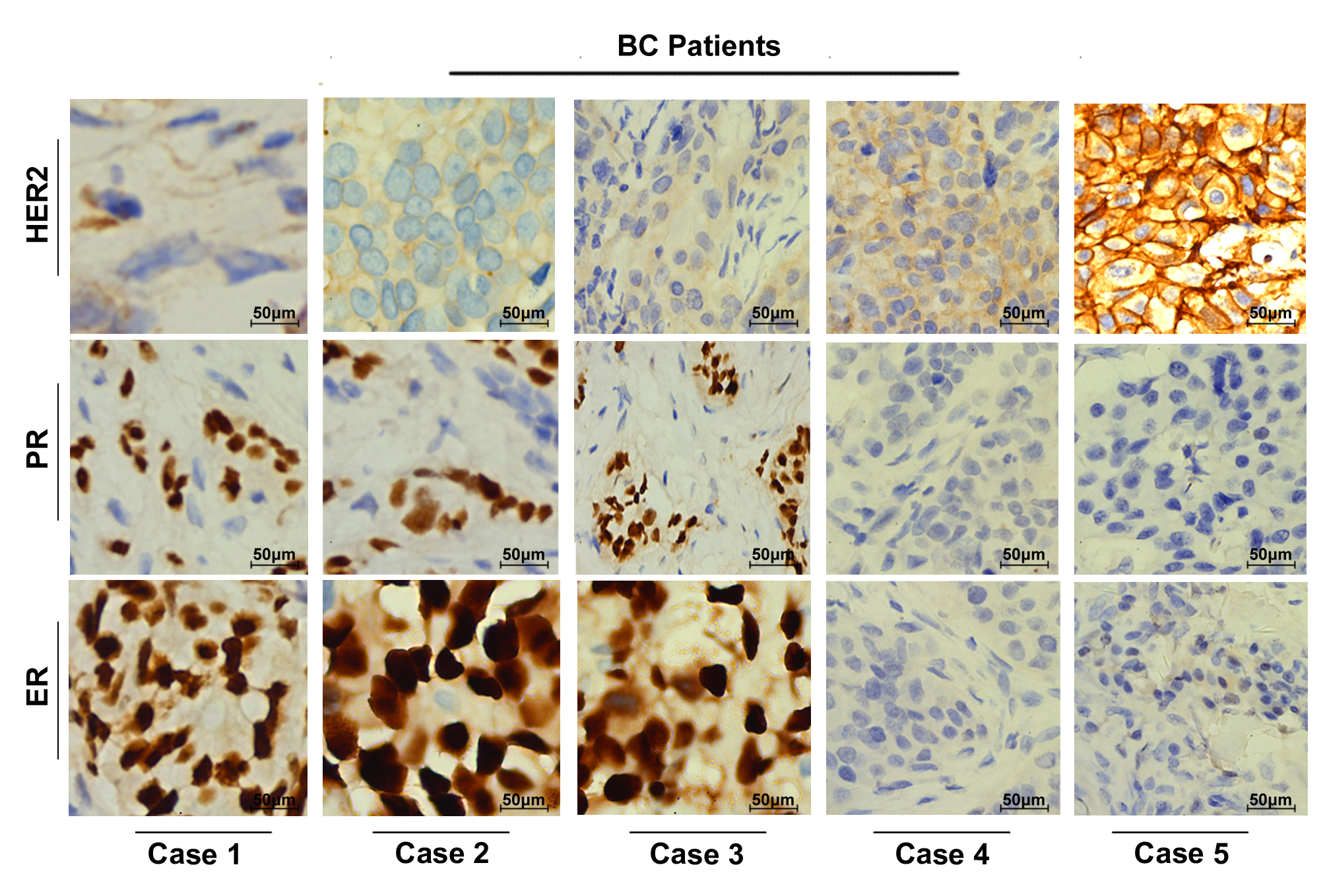

Supplement: Supplementary file 2 — Figure S1. Expression of ER, PR, HER2 in 5 primary BC samples. Three primary BC samples were clinically defined as ER+/PR+/HER2− by IHC. Two primary BC samples were clinically defined as TNBC and ER−/PR−/HER2+ by IHC. Case1:HER2−ER+PR+ Case2:HER2−ER+PR+ Case3:HER2−ER+PR+ Case4:HER2−ER−PR−(TNBC) Case5: HER2+ER−PR− Scale bar: 50 μm. (TIF 3702 kb) [file 12885_2018_4717_MOESM2_ESM.tif]
